# Revisiting the Object‐Processing Paradigm in the Study of Gaze Cues: What Two Decades of Research Have Taught Us About Infant Social Learning

**DOI:** 10.1111/infa.70007

**Published:** 2025-02-25

**Authors:** Christine Michel, Maleen Thiele

**Affiliations:** ^1^ SRH University of Applied Sciences Heidelberg Gera Germany; ^2^ Max Planck Institute for Evolutionary Anthropology Leipzig Germany

**Keywords:** gaze cue, infancy, memory, object processing, review

## Abstract

Infants are highly sensitive to social stimuli from early on in ontogeny. Social cues, including others' gaze, not only capture and guide infants' attention, but also modulate the efficiency in which the infant (brain) encodes and recognizes information. Over the last two decades, the novelty preference based object‐processing paradigm has been instrumental in investigating this phenomenon experimentally. This paper offers a comprehensive review and critical evaluation of methodological aspects and empirical findings from previous research using this paradigm to study the influence of (non‐)social cues on infants' object processing. We highlight the critical role of methodological details and discuss influential factors such as eye contact, infants' object‐directed attention, naturalistic environments, and potential neural correlates associated with enhanced object encoding. A comprehensive review table summarizes key methodological details from previous studies to assist researchers in making informed decisions when designing future studies. We conclude that the object‐processing paradigm has proven to be an effective method with high potential for future research disentangling the influence of fine‐grained factors on infants' object memory.

## 1 General Introduction

Acquiring knowledge about objects is a fundamental developmental task for infants, relying on their ability to process, memorize, and recognize novel information. Other people, and the cues they provide, play a key role in influencing these processes and facilitating social learning. Around 20 years ago, Reid et al. (2004) published their seminal paper on the influence of adult's gaze cues on infants' object memory applying the novelty preference based object‐processing paradigm. Many studies followed up on this initial work. Using adaptations of the original paradigm, this research yielded important insights into the ontogeny of social learning demonstrating that, well before engaging in imitative behaviors or word learning, infants show foundational competencies to learn from others about novel objects. The simplicity and adaptability of the object‐processing paradigm make it a valuable and versatile tool with high potential for studying infant social learning comprehensively. However, as in many developmental research paradigms, reliable and valid conclusions hinge on meticulous methodology. Careful considerations are essential for ensuring an accurate interpretation of findings and drawing broader inferences.

### 1.1 Aims and Structure of This Review

This review aims to summarize and examine the methodological adaptations of the object‐processing paradigm since its initial application, and synthesize insights gleaned from two decades of research employing this paradigm to investigate early object learning in social contexts. To distinguish this review from related research areas, we focus on studies investigating the influence of gaze cues and non‐social controls on the processing and recognition of *visual* object features in (mostly) preverbal infants. More complex social learning phenomena requiring more sophisticated cognitive operations, like word learning or action imitation, are not included. Methodologically, we focus on one specific paradigm measuring infants' object memory through their novelty response (“object‐processing paradigm” in this article). Studies employing other methods, for example, attentional cueing, habituation, or violation‐of‐expectation (Margoni, Surian, and Baillargeon 2023; Paulus 2022; Stahl and Kibbe 2022) and research using the object‐recognition paradigm to study emotional attribution (e.g., Hoehl et al. 2008; Hoehl and Striano 2008) are not part of this review.

This article is structured as follows. First, we outline the core psychological assumptions of the object‐processing paradigm and describe initial studies using the paradigm to investigate the role of gaze cues on infants' object learning. Next, we provide a comprehensive review of studies employing this paradigm, highlighting methodological variations and key insights into infant social learning, including both traditional and novel perspectives. Finally, we critically discuss the limitations of the paradigm and propose future research directions to advance our understanding of infant object processing and social learning.

### 1.2 Psychological Assumptions of the Paradigm

The encoding of information represents a core feature of memory formation and learning and refers to the perception and first registration of a memory (Wojcik 2013). In the first year of life, this capacity represents a crucial milestone, as storage and retrieval of information are foundational for more complex forms of learning. From a broader developmental perspective, encoding represents a temporary and premature learning stage, as it refers to relatively unconsolidated memory traces (Davis et al. 2009). Storing memory traces long‐term requires stabilization and integration processes, involving strengthening and connecting of initially encoded information, which is challenging for young infants and not measured with this paradigm (Bauer et al. 2011).

**FIGURE 1:**
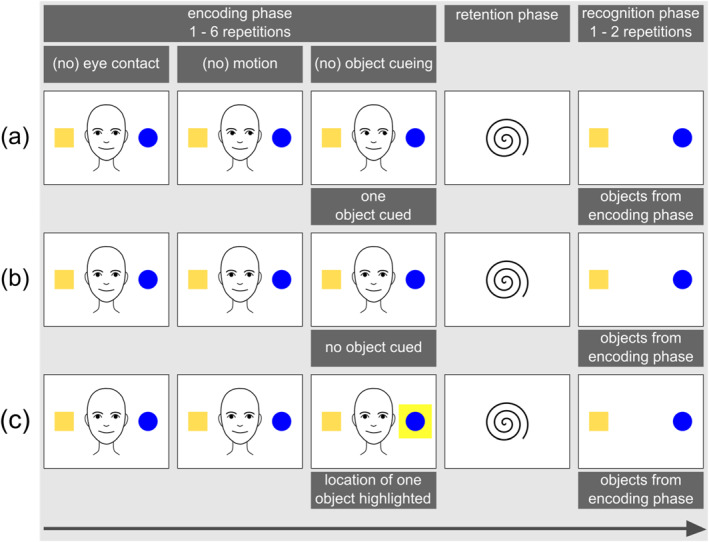
Schematic illustrations of trial procedures used in behavioral studies depicting two objects during the encoding phase and two objects during the recognition phase with (a) one object being cued, (b) no object being cued, and (c) the location of one object being highlighted.

**FIGURE 2:**
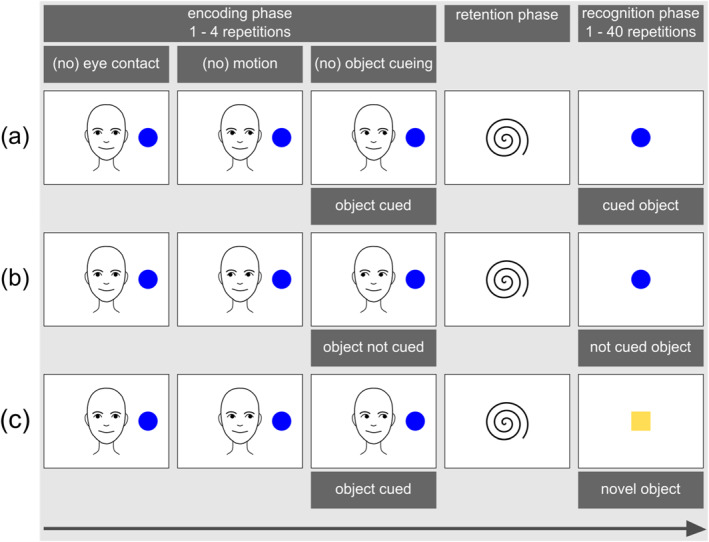
Schematic illustrations of trial procedures used in neurophysiological studies depicting one object during the encoding phase and one object during the recognition phase with (a) the object being cued and the response towards this object being measured, (b) the object not being cued, and (c) the object being cued and the response towards a novel object being measured.

**FIGURE 3:**
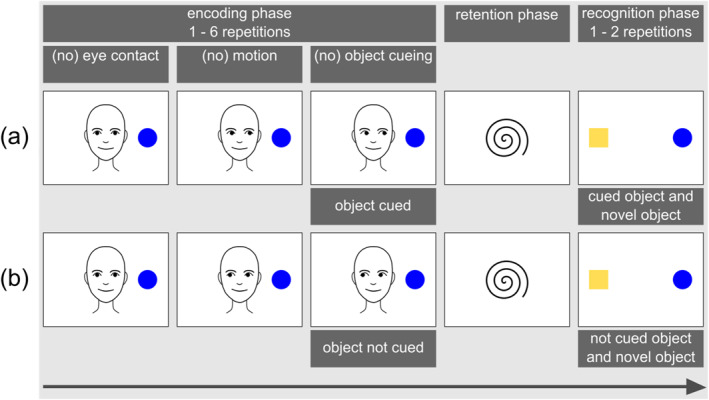
Schematic illustrations of trial procedures used in behavioral studies depicting one object during the encoding phase and two objects during the recognition phase with (a) the object being cued and (b) the object not being cued. In some studies featuring only one object, object and actor position was reversed with the object displayed in the center and the actor laterally.

Instead of directly measuring the (neural) building of this memory trace, the object‐processing paradigm takes an indirect approach, relying on infants' tendency to recall and recognize previously encoded perceptual information. More specifically, the procedure of the paradigm typically involves three phases (see Figures 1, 2, 3): (1) an initial encoding phase where the infant is exposed to a target object in a given context and potentially processes and establishes a memory trace of this object, (2) a retention phase featuring a blank screen or an attention getter, and (3) a subsequent recognition phase where the infant's recognition of the target object is measured through their novelty preference—either in response to the target object, or in contrast to another, often novel object. Infants' novelty preference in the recognition phase is used as indirect evidence of their memory for the previously seen target object, and of the effectiveness in which perceptual surface information of this object have been processed during initial exposure.

In the context of this review, the object‐processing paradigm has been used to investigate the influence of (social) cues on infants' encoding and subsequent recognition of a novel object. If a cue guided the infant's attention to a displayed target object during the encoding phase, and if this cueing facilitated their encoding of this object, it was assumed that this object would become familiar to the infant. When encountering it again in the subsequent recognition phase infants were expected to recognize this object and to demonstrate a novelty preference by orienting their attention toward the novel object. This assumption aligns with evidence showing that full stimulus encoding in infants leads to decreased attention to this stimulus and a preference for novel stimuli, while partial encoding is associated with a familiarity preference in infants (Fantz 1964; Pascalis and de Haan 2003; Rose et al. 1982; Rose, Feldman, and Jankowski 2004). Conversely, if a cue did not enhance an infant's object encoding, no memory trace of the object would be established. In this case, it was expected that the object would not be recognized in the recognition phase, resulting in no differential attention between both the unprocessed target object and novel object (no novelty preference).

Depending on the method and design of a study, researchers have employed different dependent variables for measuring infants' novelty response during the recognition phase (Reynolds 2015). On the neural level, event‐related potentials (ERPs) like the positive slow wave (PSW) or the negative central (NC) component have been used. PSW activity appears approximately 800–1000 ms after stimulus onset and has been related to stimulus encoding and memory updating processes in infants (Webb, Long, and Nelson 2005). An increased PSW during a potential phase of recognition therefore hints at an incomplete preceding encoding process. The NC component occurs approximately 300–700 ms after stimulus onset. It reflects general attentional arousal and orienting to salient stimuli (Richards 2003), and is related to the early development of memory processes (Courchesne, Ganz, and Norcia 1981). An increased NC component can arise when seeing a novel or unfamiliar object (Reynolds and Richards 2005) which—following the logic of the object‐processing paradigm—has not been (completely) encoded during the preceding encoding phase. Both components have been shown to be enhanced in response to previously not cued objects (enhanced PSW: e.g., Reid et al. 2004; enhanced NC: e.g., Wahl et al. 2013). While these studies employed similar stimuli and timings, they yielded diverging results: Infants' evaluation of novelty was represented either in heightened attention (NC) or enhanced encoding effort (PSW). This difference in the discriminatory component cannot be explained by the movement of the cue, as both moving stimuli (e.g., Reid et al. 2004) or static images (Hoehl et al. 2012) have been shown to influence the PSW. The underlying factors that may determine the activation of one component over the other remain to date unknown. Candidate factors could relate to the attributes of a cue, object characteristics, or precise timing, but their influence needs to be determined in future studies explicitly manipulating these methodological details.

On the behavioral level, infants' novelty preference has been typically assessed by measuring their preferential touching or looking response, with the latter being retrieved by manual coding procedures or automated eye‐tracking. Here, infants' novelty preference was typically measured by relatively longer looking times to the respective other object, which was either an object appearing for the first time (i.e., completely novel object), or an object that was previously presented but not highlighted by any cue and, thus, lacking a memory trace (Rose et al. 1982).

A few studies have relied on infants' touch response in a manual choice task to measure their preference (see column “dependent measure” in the OSF table). However, this task was typically combined with a preceding preferential looking test. This sequential design may have introduced confounding factors, as infants' familiarity with the objects could have been influenced by their prior visual exposure. Consequently, the subsequent manual choice response may have not solely reflected their novelty preference but could have been driven by other factors, such as their motivation to play with the object or explore its haptic features. Given these potential confounds, the validity of manual choice tasks as a measure of infant object recognition remains uncertain.

### 1.3 How It Started: The First Studies Applying the Object‐Processing Paradigm to Study Initial Memory Stages of Infants' Object Learning in a Social Context

In their original study, Reid et al. (2004) presented infants with pictures featuring an object together with a female actor unfamiliar to the infant (see Figure 2a,b for a schematic illustration). During the encoding phase (“cueing phase” in Reid et al. 2004), the actor looked in the direction of the infant before shifting her gaze to her right or left side, resulting in an object‐directed look or a look away from the object. Following a centralizing attention‐getting animation, the object reappeared in the recognition phase, presented in the center of the screen. Infants' brain activity was measured in response to this object. The authors found an enhanced PSW for previously not cued objects, which they interpreted as the gaze cue guiding infants' attention to the target object and facilitating their encoding of the cued but not the not cued object.

In 2005, Reid & Striano published a study using an adapted version of their paradigm, relying on looking times as the dependent behavioral measure (see Figure 1a for a schematic illustration). During the encoding phase, infants were again presented with pictures featuring a female actor, but this time two similarly looking objects were presented, one on each side of the actor. Again, the actor first looked at the infant before shifting her gaze to one side, cueing one of the two objects with her gaze while averting her gaze from the other (not cued) object. In the recognition phase, both objects reappeared in a paired‐preference test and infants' looking times to both objects were measured. Infants looked longer to the previously not cued object compared to the previously cued object, which the authors interpreted as confirmatory evidence that gaze cues to an object selectively facilitate infants' encoding of this object compared to a not cued object. The two above described studies (Reid et al. 2004; Reid and Striano 2005) exemplarily illustrate that adjustments in the paradigm were necessary to address the peculiarities of the methods being used. For the ERP measure, it was required to present the target object alone to isolate immediate brain responses to the cued versus the not cued object, whereas the looking time study employed a paired‐preference test during the recognition phase, presenting both objects simultaneously.

## 2 Literature Review

The object‐processing paradigm has been utilized with many methodological variations since its initial application. In Table 1 we summarize key characteristics of the studies included in our review. A more detailed breakdown of study characteristics and methodological details for each paradigm phase can be found in the Supporting Information S1 and on the Open Science Framework (OSF, https://osf.io/ysrje). Figures 1, 2, 3 illustrate variations in the procedural aspects of the studies reviewed in this article.

**TABLE 1:** Overview of the key characteristics of the reviewed studies.

Authors	Exp.	Age range	Study design	Conditions	Cue	Dependent variable	Method	Main results
Cleveland, Schug and Striano 2007	1	5.05–6.06	Figure 3a	(1) Eye contact and cued object, (2) no eye contact and cued object	Human: Gaze and head	NPS ((LT to novel/novel + familiar object) × 100)	Manual coding	No sig. difference in NPS between eye contact and no eye contact condition
	2	7.01–8.00	Figure 3a	(1) Eye contact and cued object, (2) no eye contact and cued object	Human: Gaze and head	NPS ((LT to novel/novel + familiar object) × 100)	Manual coding	Sig. increased NPS in eye contact condition versus no eye contact condition
Cleveland and Striano 2007	1 & 2							Sig. age × condition interaction (see rows below)
	1	4.06–5.06	Figure 3a	(1) Eye contact and cued object, (2) no eye contact and cued object	Human: Gaze and head	NPS ((LT to novel/novel + familiar object) × 100)	Manual coding	No sig. difference in NPS between eye contact and no eye contact condition
	2	8.17–9.10	Figure 3a	(1) Eye contact and cued object, (2) no eye contact and cued object	Human: Gaze and head	NPS ((LT to novel/novel + familiar object) × 100)	Manual coding	Sig. increased NPS in eye contact condition versus no eye contact condition
Cleveland and Striano 2008	1 & 2							Sig. main effect of age (18‐month > 14 months), sig. age × condition interaction (see rows below)
	1	13.22–14.14	Figure 3a	(1) Eye contact and cued object, (2) no eye contact and cued object	Human: Gaze and head	NPS ((LT to novel/novel + familiar object) × 100)	Manual coding	No sig. difference in NPS between eye contact and no eye contact condition
	2	17.16–18.14	Figure 3a	(1) Eye contact and cued object, (2) no eye contact and cued object	Human: Gaze and head	NPS ((LT to novel/novel + familiar object) × 100)	Manual coding	Sig. increased NPS in eye contact condition versus no eye contact condition
Hoehl et al. 2012	1	4.02–4.25	Figure 2a,b	(1) Object cued by caregiver, (2) object not cued by caregiver, (3) object cued by stranger, (4) object not cued by stranger	Human: Gaze	NC: Mean amplitude, peak amplitude PSW: Mean amplitude	EEG	No sig. difference in mean amplitude and peak amplitude of NC between cued and not cued object in caregiver and stranger condition, sig. increased mean amplitude of PSW for not cued objects versus cued objects in caregiver condition
Hoehl, Wahl and Pauen 2014	1	4.00–4.30	Figure 1a	(1) One object cued and one object not cued by gaze, (2) one object cued and one object not cued by head	Human: Gaze or head	Relative LT to each object (LT to object/LT to screen)	Eye‐tracking	Sig. increased relative LT to not cued objects versus cued objects
	2	4.00–4.29	Figure 2a,b	(1) Object cued by gaze, (2) object not cued by gaze, (3) object cued by head, (4) object not cued by head	Human: Gaze or head	NC: Peak amplitude	EEG	Sig. increased NC peak amplitude for not cued objects versus cued objects
Ishikawa et al. 2019	1 & 2							Sig. higher frequency of first touches of cued object in initiating joint attention (exp.1) versus responding to joint attention condition (exp.2)
	1	11.06–12.08	Figure 3a,b	After object appearance on screen (mock initiating joint attention): (1) object cued, (2) object not cued	Human: Gaze	Percentage of relative LT, first touch in manual choice task	Eye‐tracking, manual coding	Sig. above‐chance looking preference for not cued object, sig. first touch choice of cued object versus not cued object
	2	9.04–12.04	Figure 3a,b	Before object appearance on screen (mock responding to joint attention): (1) object (location) cued, (2) object (location) not cued	Human: Gaze	Percentage of relative LT, first touch in manual choice task	Eye‐tracking, manual coding	No sig. above‐chance looking preference for any object, sig. first touch choice of not cued object
Itakura 2001	1	9–13 (days not specified)	Figure 1b	(1) No object cued (control)	No cue	Absolute LT to each object	Manual coding	No sig. difference in absolute LT between both not cued objects
	2	9–13 (days not specified)	Figure 1a	(1) One object cued and one object not cued	Human: Gaze and pointing gesture	Absolute LT to each object	Manual coding	Sig. longer absolute LT at cued object versus not cued object
	3	9–13 (days not specified)	Figure 1c	(1) One object cued and one object not cued	Target object blinking	Absolute LT to each object	Manual coding	No sig. difference in absolute LT between cued object and not cued object
Kopp and Lindenberger 2011	1	Session 1: 8.16–9.09 Session 2: 8.23–9.16	Figure 2a,c	(1) High joint attention (eye contact, pointing, IDS) and cued object, (2) low joint attention (no eye contact, no pointing, prerecorded IDS) and cued object	Human: Gaze, head and pointing gesture	N1: Peak amplitude, peak latency PB: Peak amplitude, peak latency NC: Peak amplitude, peak latency PSW: Mean activity	EEG	No sig. difference in N1 peak amplitude or peak latency between conditions, sessions, and objects, sig. increased PB peak amplitude for familiar objects in session 2 versus session 1 in low joint‐attention condition, sig. increased NC peak amplitude for novel versus familiar objects in session 2 independent of condition, sig. increased NC peak latency for novel versus familiar objects, increased PSW mean activity for familiar versus novel objects in session 1 in high joint‐attention condition
Michel, Pauen, and Hoehl 2017	1 & 2	4.0–4.31						Sig. experiment × cue × object position interaction (see rows below)
	1		Figure 1a	(1) One object cued and one object not cued	schematic eyes: Gaze	Relative LT to each object (LT to object/LT to screen)	Eye‐tracking	Sig. increased relative LT to not cued objects versus cued objects
	2		Figure 1a	(1) One object cued and one object not cued	schematic eyes: Gaze	Relative LT to each object (LT to object/LT to screen)	Eye‐tracking	Sig. increased relative LT to not cued objects versus cued objects in trials with same position
Michel et al. 2019	1	4.00–4.30	Figure 2a,b	(1) Cued object, (2) not cued object	Turning cuboid with checkerboard pattern	NC: Mean amplitude PSW: Mean amplitude	EEG	No sig. difference in mean NC amplitude and mean PSW amplitude between cued objects and not cued objects
	2	4.01–4.29	Figure 2a,b	(1) Cued object, (2) not cued object	Turning cuboid with schematic eyes	NC: Mean amplitude PSW: Mean amplitude	EEG	No sig. difference in mean NC amplitude between cued objects and not cued objects, sig. increased mean PSW amplitude in response to not cued objects versus cued objects
Michel, Matthes and Hoehl 2024	1	9.00–10.17	Figure 3a	(1) Ostensive (eye contact, IDS, infant name) and cued object, (2) non‐ostensive (no eye contact, adult‐directed speech, no infant name) and cued object	Human: Gaze and head	NPS (LT to novel/novel + familiar object)	Manual coding	No sig. difference in NPS between ostensive and non‐ostensive condition
Okumura et al. 2013b	1	11.06–12.04	Figure 1a	(1) One object cued and one object not cued by human model, (2) one object cued and one not cued by robot model	Human or robot: Gaze and head	NPS (LT to not cued/cued + not cued object), first touch in manual choice task	Eye‐tracking, manual coding	Sig. increased NPS in human condition versus robot condition, sig. above‐chance NPS in human but not robot condition, sig. first touch choice of cued object in human but not robot condition
	2	11.06–12.05	Figure 1a	(1) One object cued and one object not cued	Robot: Head	NPS (LT to not cued/cued + not cued object), first touch in manual choice task	Eye‐tracking, manual coding	No sig. above‐chance NPS, no sig. first touch choice
	3	11.06–12.04	Figure 1c	(1) One object cued and one object not cued	Target object illuminated (blinking light)	NPS (LT to not cued/cued + not cued object), first touch in manual choice task	Eye‐tracking, manual coding	No sig. above‐chance NPS, no sig. first touch choice
Okumura et al. 2013a	1	11.05–12.05	Figure 1a	(1) Ostensive (eye contact, IDS, infant name; one object cued and one object not cued), (2) ostensive‐referential (eye contact, IDS, infant name and referencing toy verbally; one object cued and one object not cued)	Robot: Gaze and head	NPS (LT to not cued/cued + not cued object), first touch in manual choice task	Eye‐tracking, manual coding	Sig. increased NPS in ostensive‐referential condition but not ostensive condition, no sig. first touch choice
	2	11.05–12.05	Figure 1a	(1) One object cued and one object not cued	Robot: Gaze and head	NPS (LT to not cued/cued + not cued object), first touch in manual choice task	Eye‐tracking, manual coding	No sig. above‐chance NPS, no sig. first touch choice
Okumura et al. 2017	1	8.08–9.04	Figure 1a	(1) One object cued and one object not cued	Human: Gaze and head	NPS (LT to not cued/cued + not cued object)	Eye‐tracking	No sig. above‐chance NPS
	2	8.04–9.09	Figure 1c	(1) One object cued and one object not cued	Target object illuminated (blinking light)	NPS (LT to not cued/cued + not cued object)	Eye‐tracking	No sig. above‐chance NPS
Okumura et al. 2020	1, 2, & 3							Sig. differences between gaze‐only (exp.1) and IDS conditions (exp.3) and between shivering (exp.2) and IDS conditions (exp.3), no sig. difference between gaze‐only (exp.1) and shivering conditions (exp.2) (see rows below)
	1	8.06–9.04	Figure 1a	(1) One object cued and one object not cued	Human: Gaze and head	NPS (LT to not cued/cued + not cued object), first touch in manual choice task	Eye‐tracking, manual coding	No sig. above‐chance NPS, no sig. first touch choice
	2	8.04–9.07	Figure 1a	(1) One object cued and one object not cued	Human: Gaze and head	NPS (LT to not cued/cued + not cued object), first touch in manual choice task	Eye‐tracking, manual coding	No sig. above‐chance NPS, no sig. first touch choice
	3	8.07–9.05	Figure 1a	(1) One object cued and one object not cued	Human: Gaze and head	NPS (LT to not cued/cued + not cued object), first touch in manual choice task	Eye‐tracking, manual coding	Sig. above‐chance NPS, sig. first touch choice of cued object
	4	8.06–9.08	Figure 1a	(1) One object cued and one object not cued	Human: Gaze and head	NPS (LT to not cued/cued + not cued object), first touch in manual choice task	Eye‐tracking, manual coding	Sig. above‐chance NPS, sig. first touch choice of cued object
	5	8.05–9.09	Figure 1a	(1) One object cued and one object not cued	Human: Gaze and head	NPS (LT to not cued/cued + not cued object), first touch in manual choice task	Eye‐tracking, manual coding	No sig. above‐chance NPS, no sig. first touch choice
	6	8.06–9.08	Figure 1a	(1) One object cued and one object not cued	Human: Gaze and head	NPS (LT to not cued/cued + not cued object), first touch in manual choice task	Eye‐tracking, manual coding	No sig. above‐chance NPS, no sig. first touch choice
	7	8.06–9.09	Figure 1a	(1) One object cued and one object not cued	Human: Gaze and head	NPS (LT to not cued/cued + not cued object), first touch in manual choice task	Eye‐tracking, manual coding	No sig. above‐chance NPS, no sig. first touch choice
Parise et al. 2007	1	10.19–12.24	Figure 3a	(1) Cued object with IDS, (2) cued object without voice	Human: Gaze and head	Absolute LT to each object	Manual coding	First recognition trial: Sig. increased absolute LT to novel object following cueing videos involving IDS versus cueing videos lacking voice, second recognition trial: No sig. condition difference in absolute LT
Parise et al. 2008	1	5.01–6.03	Figure 2a	(1) Eye contact and cued object, (2) no eye contact and cued object	Human: Gaze and head	NC: Peak amplitude	EEG	Increased NC peak amplitude on left fronto‐central electrodes for objects cued with eye contact versus objects cued without eye contact
Pickron, Fava and Scott 2017	1	Mean = 5.19	Figure 1a	(1) Familiar sex and other race and cued object, (2) less familiar sex and other race and cued object, (3) familiar sex and own race and cued object, (4) less familiar sex and own race and cued object	Human: Gaze	Absolute LT to each object	Eye‐tracking	Sig. sex × object type interaction: Sig. Longer absolute LT to not cued objects versus cued objects in familiar sex conditions (no effect in less familiar sex conditions), sig. race × sex × object type interaction: Longer absolute LT to not cued objects versus cued objects in own‐race and familiar‐sex conditions
	2	Mean = 10.06	Figure 1a	(1) Familiar sex and other race and cued object, (2) less familiar sex and other race and cued object, (3) familiar sex and own race and cued object, (4) less familiar sex and own race and cued object	Human: Gaze	Absolute LT to each object	Eye‐tracking	Sig. sex × object type interaction: Sig. Longer absolute LT to not cued objects versus cued objects in familiar sex conditions (no effect in less familiar sex conditions), sig. race × sex × object type interaction: Longer absolute LT to not cued objects versus cued objects in own‐race and familiar‐sex conditions
Reid and Striano 2005	1	15–17 (days not specified)	Figure 1a	(1) One object cued and one object not cued	Human: Gaze	Absolute LT to each object	Manual coding	Sig. increased absolute LT to not cued objects versus cued objects
	2	14.5–18 (days not specified)	Figure 1b	(1) No object cued	Human: No cue provided	Absolute LT to each object	Manual coding	No sig. differences in absolute LT to both not cued objects
Reid et al. 2004	1	15–17 (days not specified)	Figure 2a,b	(1) Cued object, (2) not cued object	Human: Gaze	PSW: Peak amplitude	EEG	Sig. increased slow wave peak amplitude to not cued objects versus cued objects
Striano et al. 2006	1	11.27–13.0	Figure 3a	(1) Eye contact and cued object, (2) no eye contact and cued object	Human: Gaze and head	NPS ((LT to novel/novel + familiar object) × 100)	Manual coding	No sig. differences in NPS between eye contact and cued object and no eye contact and cued object condition
	2	8.11–9.22	Figure 3a	(1) Eye contact and cued object, (2) no eye contact and cued object	Human: Gaze and head	NPS ((LT to novel/novel + familiar object) × 100)	Manual coding	Sig. increased NPS in eye contact and cued object as versus no eye contact and cued object condition
Theuring, Gredebäck and Hauf 2007	1	Not reported	Figure 1a	(1) One object cued and one object not cued	Human: Gaze and head	NPS ((LT to novel/novel + familiar object)*100), FPS ((LT to familiar/novel + familiar object) × 100)	Eye‐tracking	first recognition test trial: Sig. increased NPS versus FPS, second recognition test trial: No sig. difference between NPS and FPS
Thiele et al. 2021	1	9.0–10.0	Figure 3a,b	(1) Eye contact and cued object, (2) no eye contact and cued object, (3) eye contact and no cue at object, (4) no eye contact and no cue at object	Two humans: Gaze and head	NPS (LT to novel/novel + familiar object)	Eye‐tracking	Sig. Eye contact × object cueing interaction: Sig. increased NPS in eye‐contact and cued object condition versus all other conditions
	2	9.0–10.0	Figure 3a,b	(1) Eye contact and object cued, (2) no eye contact and object cued, (3) eye contact and object not cued, (4) no eye contact and object not cued	Human: Gaze and head	NPS (LT to novel/novel + familiar object)	Eye‐tracking	Sig. eye contact × object cueing interaction: Sig. increased NPS in eye‐contact and cued object condition versus all other conditions
Wahl, Marinović and Träuble 2019	1	4.10–4.30	Figure 1a	(1) One object cued and one object not cued	schematic eyes: Gaze	Absolute LT to each object	Eye‐tracking	Sig. increased absolute LT to not cued objects versus cued objects
	2	4.12–4.30	Figure 2a,b	(1) Cued object, (2) not cued object	schematic eyes: Gaze	NC: Mean amplitude PSW: Mean amplitude	EEG	Encoding phase: Sig. increased PSW for cued objects versus not cued objects, recognition phase: Sig. increased NC activity for not cued versus cued objects
Wahl et al. 2013	1	4.00–4.31	Figure 1a	(1) One object cued and one object not cued	Human: Gaze and head	Relative LT to each object (LT to object/LT to screen)	Eye‐tracking	Sig. increased relative LT to not cued objects versus cued objects
	2	4.00–4.29	Figure 2a,b	(1) Cued object, (2) not cued object	Human: Gaze and head	PB: Mean amplitude, peak amplitude, peak latency NC: Mean amplitude, peak amplitude, peak latency PSW: Mean amplitude	EEG	Sig. increased PB mean amplitude for cued objects versus not cued objects, sig. increased PB peak amplitude for cued objects versus not cued objects, sig. increased NC mean amplitude for not cued objects versus cued objects, sig. increased NC peak amplitude for not cued objects versus cued objects
	3	4.02–4.30	Figure 1a	(1) One object cued and one object not cued	Turning car	Relative LT to each object (LT to object/LT to screen)	Eye‐tracking	No sig. difference in relative LT to cued objects and not cued objects
	4	4.02–4.28	Figure 2a,b	(1) Cued object, (2) not cued object	Turning car	PB: Mean amplitude, peak amplitude, peak latency NC: Mean amplitude, peak amplitude, peak latency PSW: Mean amplitude	EEG	No sig. difference in PB mean amplitude, PB peak amplitude, PB latency, NC mean amplitude, NC peak amplitude, NC latency between cued objects and not cued object, sig. increased PSW mean amplitude for not cued objects versus cued objects in only one specific time window

*Note:* “Study Design” refers to the specific sub‐section (a, b, or c) of the respective Figure (Figures 1, 2, or Figure 3). “Method” refers to the methodological approach extracting the dependent variable. NPS = novelty preference score. LT = looking time. Event‐related potentials: PSW = positive slow wave, NC = negative central component, N1 = early negative component, PB = positive before component. A more detailed breakdown of study characteristics and methodological details for each paradigm phase can be found in the Supplementary Table S1 and on the Open Science Framework (OSF, https://osf.io/ysrje).

### 2.1 Methodological Variations and Considerations in the Object‐Processing Paradigm

A critical factor influencing the interpretability of the object‐processing paradigm is the degree to which infants perceive objects as novel or familiar during the recognition phase. To help infants discriminate novel from familiar objects, methodological considerations are crucial. For example, the pairing of objects in terms of their visual similarity needs to be considered (column “general criteria for object matching” and “criteria for object pairs” in the OSF table), and the background color should be consistent across encoding and recognition phases (column “color of background in recognition phase compared to encoding phase” in the OSF table) (Robinson and Pascalis 2004). Moreover, given that infants' visual skills and capacities like memory and processing speed improve with age (Colombo 2001; Pelphrey et al. 2004; Reznick et al. 2004), an inappropriate timing for a tested age group may lead to the absence of a preference, which is difficult to interpret: If encoding or recognition phase are too long for older infants, they may quickly encode the cued *and* not cued object (column “total duration of recognition phase per object (pair)” in the OSF table). Shorter presentation times, in turn, may disadvantage younger infants' performance. Furthermore, infants may lose the memory trace of a processed object if the retention interval is too long (column “duration of retention phase” in the OSF table). Aligning with this assumption, previous studies comparing multiple age groups revealed inconsistent results. For example, Cleveland, Schug, and Striano (2007) and Cleveland and Striano (2007) found a novelty preference for the novel object only in 5‐ and 7‐month‐olds, but not in 4‐ and 9‐month‐olds. Similarly, Striano et al. (2006) reported that only 9‐month‐olds, not 12‐month‐olds, showed increased object encoding following joint attention in an interactive setting with an experimenter, while Ishikawa et al. (2019) found no evidence for facilitated object encoding in their screen‐based “responding to joint attention” condition in 10‐ to 12‐month‐olds (but see Cleveland and Striano 2008; Okumura et al. 2013a, 2013b). To accommodate variations in cognitive abilities, developing individual‐based presentation times and measurement periods could be a promising strategy to explore in future studies.

It remains an open question how long the memory effect lasts. While some evidence suggests short‐term effects (e.g., Theuring, Gredebäck, and Hauf 2007), long(er)‐term memory effects are rarely studied and require further investigation (Kopp and Lindenberger 2011; Parise et al. 2007). Another open question concerns the precise interpretation of most dependent variables, especially preferential looking times. It remains, for example, unclear whether a linear increase in the preference score directly translates to a linear increase in infants' processing and their recognition performance. Relatedly, it remains to be studied whether different preference scores, when observed above chance‐level, indicate a qualitatively different processing depth. Investigating these questions would be crucial for our understanding of the memory building process, as well as for evaluating the contributions and constraints of the paradigm in uncovering those processes.

Beyond novelty and familiarity, several other factors can potentially influence infants' response. For instance, intrinsic biases or individual preferences for certain objects might exist, independent of the encoding and recognition process. Additionally, the salience of an object's color or size might inherently bias attention. A strategic pairing of objects or explicitly pre‐testing object preferences could help overcome this potential confound (Michel, Pauen, and Hoehl 2017; Michel, Matthes, and Hoehl 2024). Furthermore, Becchio, Bertone, and Castiello (2008) proposed that social cues like another person's gaze may enrich an object with additional information like the looker's affective reaction to the object. This, in turn, may influence the infants' own response to the object, such that they approach or avoid the object. Thus, a seemingly simple novelty or familiarity preference may sometimes reflect more than the mere status of encoding, for example, a reaction toward a socio‐affective meaning transferred to the object (Snyder, Blank, and Marsolek 2008).

Another methodological variation relates to the use of static and dynamic stimuli to operationalize gaze cueing during the encoding phase. While the majority of reviewed studies employed dynamic stimuli, such as dynamic videos or real‐life interactions, some studies utilized a series of still images to simulate apparent motion (e.g., Hoehl et al. 2012; Ishikawa et al. 2019). A comparison of the results across these studies suggests that the facilitating effect of gaze cues on infant object processing does not depend solely on explicit motion. Rather, it appears to be robust, regardless of whether the cue is presented dynamically or statically, as long as the relevant social cues are present (infant‐directed and object‐directed gaze, as discussed in section “The Influence of Social Cues”).

### 2.2 Infants' Object Processing in Screen‐Based Studies: What Are Effective Cues?

#### 2.2.1 The Influence of Social Cues

Even though infants rarely encounter isolated gaze within their natural environment (Reid and Striano 2007), the influence of gaze cues has taken on a special role in the literature. Infants encounter eye contact and gaze‐based communication from early on (with cross‐cultural variation, Kärtner, Keller, and Yovsi 2010; W. J. Schmidt et al. 2023). Moreover, the visual modality is one of the first through which infants can signal communicative intent and guide the attention of others, before targeted touching or talking abilities emerge (Raz and Saxe 2020). In addition to capturing infants' attention (see Grossmann 2017; Hoehl et al. 2009 for reviews) and increasing it (Bánki et al. 2024), the gaze of others can shift infants' own attentional focus to content outside of the interpersonal dyad. This can happen covertly (e.g., Farroni et al. 2003; Hood, Willen, and Driver 1998) and around 4–6 months also overtly, when infants begin to follow others' gaze direction with their own gaze (see Del Bianco et al. 2019 for a review on findings and theories on gaze following). Moreover, during the second half of the first year of life, infants' maturing capacities to selectively attend and respond to others' gaze cues enables them to *coordinate* their attention with others to objects (Striano and Reid 2006) with an increasing awareness of interpersonal sharedness (“joint attention”, Siposova and Carpenter 2019).

Several socio‐cognitive accounts have claimed that infants' early sensitivity to others' gaze provides them with access to social cues and facilitates social interaction and early object learning (Csibra and Gergely 2006, 2009; Reid and Striano 2007). The studies reviewed here have contributed important empirical evidence supporting this claim. The majority of these studies investigated the influence of social cues (mainly head and/or gaze cues) on infants' object encoding using screen‐based experimental designs. Cumulative evidence suggests that, by 4 months of age, infants show signs of facilitated encoding of objects when cued by the head and gaze direction of an unfamiliar person with neutral facial expression. Effects in this very young age group have been documented on the behavioral as well as on the neural level (e.g., Reid et al. 2004; Reid and Striano 2005), hinting on an equal sensitivity of ERPs and looking times in this paradigm. In addition, studies applying both methods revealed identical findings across ERPs and looking times, suggesting a similar sensitivity of both methods for detecting the facilitating effect of gaze cues on object processing (Hoehl, Wahl, and Pauen 2014; Wahl et al. 2013; Wahl, Marinović, and Träuble 2019). Looking times only allow for a broad measure of infants' novelty response. In contrast, the ERP components mainly analyzed in the context of the object‐processing paradigm, allow for a specification of the processes underlying this broad novelty response, namely heightened attention (NC) or enhanced encoding effort (PSW).

Some studies contrasted cueing conditions with control conditions featuring a face but no object cueing (Itakura 2001; Reid and Striano 2005). Similar results were found for isolated gaze cues within the context of a face, that is, eyes turning into one direction with the head remaining stationary, using neural measures (Hoehl, Wahl, and Pauen 2014; Reid et al. 2004; Reid and Striano 2005).

Contrary to previous findings regarding infants' gaze following behavior (e.g., Michel et al. 2021; Tomasello et al. 2007), 4‐month‐olds can effectively encode objects based on head cues alone, with the eyes of the cue provider remaining to look at the infant (Hoehl, Wahl, and Pauen 2014). Even completely isolated eyes without the context of a face (operationalized as black dots on white backgrounds) facilitate 4‐month‐olds’ object encoding, but only if the contrast polarity of the eyes is intact, not if it is reversed (Michel, Pauen, and Hoehl 2017; Wahl, Marinović, and Träuble 2019).

In summary, these studies demonstrate that head and/or gaze cues provided by an unfamiliar social partner facilitate the encoding of visual object features from 4 months onwards. In a study by Hoehl et al. (2012), this response was directly compared to a cueing situation involving a person of *high* social relevance. In 4‐month‐old infants, previously not cued objects elicited a more pronounced PSW component as compared to previously cued objects, but only if the caregiver, not if a stranger provided the gaze cue. Another study has highlighted the importance of caregivers (Itakura 2001). In contrast to an expected novelty preference, however, 9‐ to 11‐month‐olds in this study attended *longer* to objects previously cued by the mother's gaze direction and pointing gesture compared to a not cued object. One possible explanation for this reverse result pattern (enhanced attention to the previously *cued* object) could be the high socio‐emotional relevance of the cue given that it was embedded within a live social interaction potentially charging the object with additional meaning. A study by Pickron, Fava, and Scott (2017) furthermore showed that 5‐ and 10‐month‐olds take sex and ethnicity into account when learning from others' gaze cues. Infants showed signs of facilitated object encoding for partners with the same ethnicity as the infant and the same sex as their primary caregiver. In combination, these findings suggest that already young infants can use others' gaze cues selectively, depending on their social relevance or similarity with the infant.

Despite accumulating evidence that head and gaze cues facilitate infants' object encoding, it remains unclear what other (social) cues elicit similar effects, within the visual modality and beyond. Investigating this question would be a highly relevant future avenue considering a growing body of literature emphasizing that a sole focus on visual forms of communication neglects the within and cross‐cultural diversity in social interaction situations children encounter (e.g., Shneidman and Woodward 2016). Based on prior studies, potential candidates could be cues known to navigate infants' attention orienting or to establish interpersonal connectedness, such as infant‐directed speech (Senju and Csibra 2008; but see Peykarjou, Wissner, and Pauen 2024), the infant's own name (Parise, Friederici, and Striano 2010), pointing gestures (Itakura 2001), contingency (e.g., Deligianni et al. 2011), biological motion (e.g., Wronski and Daum 2014), or proximity and touch (Yu and Smith 2013).

#### 2.2.2 The Influence of Lower‐Level Stimulus Characteristics and Nonsocial Cues

The social stimuli used in the previously described studies all contained some degree of motion. A possible leaner explanation for the memory effect could therefore be that stimulus motion per se guided infants' attention to the cued location and, this way, increased object encoding (Deák 2015). However, studies showing that non‐social moving cues do not influence infants' neural encoding processes to the same extent as social stimuli speak against this lower‐level explanation. For instance, in studies by Okumura and colleagues, the movement of a robot's head and gaze did not facilitate 12‐month‐olds’ object encoding unless it was accompanied by ostensive referential verbal cues (Okumura et al. 2013a, 2013b). In other studies, a turning car or a turning box with a chess pattern elicited only weak or no encoding effects (Michel et al. 2019; Wahl et al. 2013). When increasing the social characteristics of the cue, however, infants' response was comparable to their response to human gaze cues: when a turning box with schematic eyes (i.e., two black circles on a white background) (not‐) cued an object, a pronounced PSW component in response to the previously not cued object was found (Michel et al. 2019). This suggests that even a few social features, such as the line‐drawing of eyes, turn a stimulus into a meaningful cue.

### 2.3 Infants' Object Encoding in More Naturalistic Social Interactions

Most of the so far reviewed experiments have been conducted in screen‐based settings allowing for highly controlled stimulus designs, and simplified conditions for infants to detect and benefit from precisely manipulated cues (for a review see Kominsky et al. 2022). While increasing internal validity, this approach is not representative of infants' highly dynamic, real interaction experience. In naturalistic interactions, the interaction partner is present with the infant, reacting continuously to their actions and talking to them. Additionally, they use gestures in combination with gaze cues, such as pointing gestures or motionese (Nomikou, Rohlfing, and Szufnarowska 2013). Accounting for this, a growing body of research aims to complement findings from lab‐based studies with ecologically more valid insights into infants' learning by investigating their everyday experience in more naturalistic settings (Hoehl and Markova 2018; Schroer, Peters, and Yu 2024; Slone et al. 2018; Wass and Goupil 2022). Despite not being fully naturalistic, some experimental studies have used the object‐recognition paradigm in live settings, for example, with the interaction partner and/or the object being physically present off screen (column “medium of cue presentation in encoding phase”, “medium of object presentation in encoding phase”, and “medium of object presentation in recognition phase” in the OSF table). These studies revealed mixed results. For example, in a series of studies, infants were familiarized with real objects in two conditions: In a “joint attention” condition, an adult experimenter established eye contact with the infant and then shifted her gaze between the infant and a real object. In a matched “object‐only” condition, the experimenter did not establish eye contact with the infant, but instead alternated her gaze between an object and the ceiling (Cleveland, Schug, and Striano 2007; Cleveland and Striano 2007; Striano et al. 2006). Only in the joint attention condition, infants at 7 and 9 months, but not younger or older infants, showed a pronounced looking time preference for a novel object when subsequently presented next to a familiarized object.

While the interaction between experimenter and infant was more dynamic compared to screen‐based approaches in these studies, the study procedure remained precisely scripted (cf., Parise et al. 2007). Relying on a more naturalistic setting, Michel, Matthes, and Hoehl (2024) let mothers interact more freely with their 9‐month‐olds. Testing the influence of the ostensive cues eyes gaze, infant‐directed speech and calling the infant by their names, mothers were asked to present a novel object to their child while either providing a combination of all these cues (“ostensive” condition) or ‐ in a “non‐ostensive” condition ‐ talking in adult‐directed speech without saying the infant's name or looking at them. Although infants seemed to notice the difference in their mothers' interaction behavior, their group performance in a subsequent object‐recognition test showed no evidence for facilitated object processing in either of the two familiarization conditions. As discussed by the authors, potential factors contributing to the absence of an effect could be the higher complexity of the interaction situation, or a longer retention interval between familiarization and recognition due to the real‐live interaction procedure and continuously measured infant EEG.

Another line of research has applied hybrid versions of the object‐processing paradigm. For example, Cleveland and Striano (2008) presented the social partner on screen, but the objects were physically present. The facilitating effect of gaze cueing on object processing was replicated in 18‐, but not 14‐month‐olds. Conversely, in a study by Parise et al. (2008), a live experimenter familiarized 5‐month‐olds with objects presented on a screen, either in combination with eye contact or without. Contrary to the results reviewed before, infants responded with an *enhanced* NC component to objects familiarized in an interaction involving eye‐contact (but see Kopp and Lindenberger 2011 for conflicting evidence in 9‐month‐olds). However, it should be noted that object cueing was also involved in the control condition, just without eye contact. Similar results were found in the above‐mentioned study by Itakura (2001), where mothers were instructed to point and comment on one out of two line drawings on screen, or to look at them without pointing and talking (experimental control). In the recognition phase, infants aged 9–13 months looked longer to the drawing the mother had previously highlighted. Similar to screen‐based studies featuring meaningful social partners, the cueing by a real person addressing the infant might render an object as socially relevant, thereby enhancing attention to the object after recognizing it.

Summing up, the complexity of the environment (e.g., controlled lab or noisier environment), methodological details (e.g., concerning the retention interval), and the richness and dynamic nature of social cues by the interaction partner (e.g., movement, speech, gestures, facial expression) in more naturalistic setups may have an influence on infants' attention during encoding and recognition, and require systematic investigation in the future.

### 2.4 The Role of Eye Contact Between Infant and Adult for Infants' Object Encoding

According to the Natural Pedagogy account, mutual eye contact can serve as an ostensive cue signaling communicative intent to the infant and announcing upcoming referential knowledge transmission (Csibra and Gergely 2006, 2009). Aligning with this idea, some object‐processing studies have investigated whether initial eye contact before referential gaze cueing has a special influence on infants' object encoding. For example, the above described live‐interactive studies by Cleveland and colleagues revealed that 7‐ and 9‐month‐olds encoded an object superiorly when addressed through eye contact compared to no eye contact (Cleveland, Schug, and Striano 2007; Cleveland and Striano 2007; Striano et al. 2006; for similar evidence using neurophysiological measures, see Parise et al. 2008). Thiele et al. (2021) found similar effects in 9‐month‐olds when an actor was presented on screen. Additionally, eye contact alone (i.e., without subsequent referential gaze shifting) was not sufficient to elicit the effect.

Okumura et al. (2020) systematically tested the influence of eye contact along with other kinds of (non‐)ostensive cues on infants' object encoding. In a series of experiments, 9‐month‐olds saw a woman on screen shifting their gaze to one of two objects. The conditions varied regarding the amount and kind of attentional and ostensive cues provided before the gaze shift: Either the woman just looked at the infant (i.e., direct gaze only), or she provided additional attentional cues (shaking her head, beep sounds) or ostensive cues (infant‐directed speech) before turning. In a control condition, the actor did not look at the infant at all. While infants *followed* the actor's gaze in all conditions except the control condition, their *encoding* of the target object was only facilitated when the child had been addressed via infant‐directed speech. A moment of mutual gaze alone prior to an object‐directed gaze cue was not sufficient to elicit the effect. The authors concluded that the availability of ostensive context is crucial for infants' encoding of gaze‐cued objects. However, as the results by Okumura et al. (2020) partly contradict studies demonstrating successful encoding in the presence of eye contact without any additional ostensive cue (e.g., Reid et al. 2004; Reid and Striano 2005), this conclusion needs further investigation. Nevertheless, previous research, in sum, emphasizes the importance of interpersonal connectedness for unlocking the scaffolding effect of social cues on infants' learning.

### 2.5 The Role of Infant's Own Looking at the Object for Their Object Encoding

The Directed Attention Model of Infant Social Cognition (Reid and Striano 2007) suggests that social cues like head and gaze can guide infants' attention to external locations and, this way, facilitate their encoding of objects presented in this location. What the account does not specify is whether the infant's attention must be shifted *overtly* in the direction of the object, or if *covert* shifts of attention can elicit the effect, too (Posner 1980). Reviewing the methodological details and results from previous studies, it is unlikely that infants' object encoding depends on their own overt looking at the object. First, in many studies the duration of the referential cueing was too short to allow overt gaze shifts to the object (e.g., 1 second in Hoehl, Wahl, and Pauen 2014; Reid and Striano 2005; Wahl et al. 2013). In the study by Reid et al. (2004), indeed most infants kept fixating at the central actor in the encoding phase instead of overtly shifting their gaze to the cued object. Wahl et al. (2013) furthermore found no differences in infants' looking times at the cued and the not cued objects during the cueing sequence. Other studies using longer presentation times in the encoding phase (e.g., 11 s in Okumura et al. 2013b, 2013a, 2017, 2020; 20 s in Itakura 2001) showed that infants' own direct gazing at an object does not automatically lead to facilitated encoding–neither when being the result of a social response (e.g., following others' gaze), nor when elicited non‐socially through stimulus enhancement (e.g., object blinking or illumination). Although spending the same amount of time looking at objects, condition differences in infants' subsequent recognition performance have been documented across several studies (Cleveland, Schug, and Striano 2007; Cleveland and Striano 2007; Parise et al. 2008; Striano et al. 2006). In three studies, the relation between infants' looking times at the object during encoding and their subsequent recognition performance has been analyzed directly. Thiele et al. (2021) did not find a statistically significant relation between infants' own looking time at the object in the encoding phase and their preference score during the recognition phase. The preference score did not even depend on whether infants' had looked at the object at all during encoding. Okumura et al. (2017) found a positive relation between infants' proportional looking time to the cued object during the cueing period of the encoding phase and the subsequently measured recognition performance, but only when a human gaze cued the object, not when the object was highlighted by blinking. In contrast, Michel, Matthes, and Hoehl (2024) revealed a negative relation between infants' looking times at the object during encoding and their novelty preference score, implying that shorter overt attention to the object led to better recognition. The authors speculated that shorter looking times may reflect faster encoding.

In sum, this evidence speaks against the idea that overt looking at an object is a prerequisite for its enhanced encoding. As suggested by Michel, Matthes, and Hoehl (2024), it would be possible that looking times may serve as an indicator of encoding speed when giving infants sufficient time for object exploration. This idea bears the potential to identify inter‐individual differences in the processing of visual information. Future research is needed to understand the processes going on during the encoding phase (see section “Neural correlates of (Successful) Encoding of Visual Object Features”).

### 2.6 Neural Correlates of (Successful) Encoding of Visual Object Features

So far, we have focused on correlates of infants' object processing, measured by their response to previously familiarized objects. In this section, we review studies investigating neural processes *during* the encoding situation itself. There are at least four candidates that may underlie infants' encoding ‐ either independently or intertwined: PSW, alpha suppression, theta band activity and interbrain synchronization. The PSW has been related to processes reflecting stimulus encoding and memory updating processes in infants (Webb, Long, and Nelson 2005). Wahl, Marinović, and Träuble (2019) reported an enhanced PSW for objects being cued by schematic eyes during the encoding phase, potentially reflecting an enhanced encoding of these objects. However, the relation between the magnitude of the PSW during encoding and infants' subsequent recognition performance for this object was not investigated.

Suppression in the alpha frequency range has been found in infants when seeing an actor looking at an object (i.e., an intact gaze‐object relation), when perceiving a novel object after a moment of mutual gaze, and when experiencing an actor following their own gaze (live‐interactive setting: Hoehl et al. 2014; screen‐based setting: Michel et al. 2015; Rayson et al. 2019). This suggests that alpha suppression represents a neural correlate of infants' processing of gaze‐object relations and mutual gaze. Enhanced theta band activity, on the other hand, has been found to relate to infants' object encoding performance, at least when 11‐month‐olds explored objects on their own (Begus, Southgate, and Gliga 2015). It is also related to infants' attention in live social interactions (Wass et al. 2018). Vice versa, there is evidence suggesting that presenting information rhythmically in theta frequency facilitates 9‐month‐olds’ information processing (Köster, Langeloh, and Hoehl 2019).

To elucidate neural processes during encoding, infants' neural processes during encoding must be related to the subsequent outcome of this process (i.e., infants' recognition performance). To our knowledge, only Michel, Matthes, and Hoehl (2024) performed such an analysis. During a live interaction with their mother and an object, infant's brain activity was continuously measured using EEG. Afterward, infants performed an object recognition test. On the group level, no difference in infants' theta or alpha activity was found. However, higher theta activity during the encoding phase predicted infants' later object recognition performance. No such relation was found for alpha activity, strengthening the importance for theta as a neural correlate of early infant learning (Begus and Bonawitz 2020).

During the last years, there has been an increasing interest in the synchrony and interplay between neural processes within two or more interaction partners during social interactions in developmental populations (Nguyen et al. 2020). When it comes to information transfer between interaction partners, neural synchrony is considered to play a key role (Hasson et al. 2012). Studies with adults have shown that the phase of oscillatory brain activity is crucial for processing incoming information (see Peelle and Davis 2012 for a review of adult language research). It has been suggested that social signals like mutual gaze may reset the oscillatory phase in the infant and adult brain and thereby provide an optimal alignment of phase and stimulus (Leong et al. 2017; Wass et al. 2020). For instance, a hyperscanning study measuring neural activity in the infant and adult brain simultaneously has shown that infant and adult oscillatory brain activity mutually influence each other during nursery rhyme singing (Leong et al. 2017). This effect is enhanced when eye contact is present, suggesting that oscillatory neural rhythms and synchronized behaviors like mutual gaze influence each other, potentially promoting learning (Markova, Nguyen, and Hoehl 2019; but see Marriott Haresign et al. 2023 for conflicting evidence). First tentative evidence for a functional interplay between interbrain synchrony and learning performance come from studies investigating neural synchrony in relation to student's performance in instructor‐based learning setups (see Tan, Wong, and Teo 2023 for a review). However, future studies are needed to test this assumption systematically and with developmental populations measuring both, interbrain synchrony and encoding performance.

### 2.7 Novel Perspectives on Infants' Object Processing in Social Interactions

#### 2.7.1 Acknowledging Infants' Active Role in Social Learning

Traditionally, the infant has been viewed as a passive learner who strongly relies on adult guidance. In contrast, more recent perspectives have emphasized the infant's *active role* in engaging with and learning from others (Begus and Southgate 2018; Crivello, Phillips, and Poulin‐Dubois 2018; Raz and Saxe 2020). In line with curiosity‐driven learning and information seeking accounts, studies have shown that infants during the second half of the first year of life perform behaviors indicating that they actively seek information through social interaction (for an overview see Begus and Southgate 2012). Around the same age, infants increasingly signal communicative intent toward potential interaction partners, invite others to jointly attend to objects, and are sensitive to whether a partner follows their invitation (e.g., Carpenter et al. 1998; Clearfield, Osborne, and Mullen 2008; Grossmann, Lloyd‐Fox, and Johnson 2013; Ishikawa et al. 2019; Rayson et al. 2019). Other research suggests that infants' active involvement in arranging their learning material can have a supportive effect on their learning: Experimental induction of curiosity enhances 8‐month‐olds’ encoding of object features (Chen, Twomey, and Westermann 2022), and their own active toy choice helps 16‐ to 18‐month‐olds memorize its functions or labels (e.g., Begus, Gliga, and Southgate 2014; Lucca and Wilbourn 2019). Together, these findings speak to the idea that infants' object memory in social interaction contexts may be particularly enhanced when choosing an object themselves.

We are aware of only one published study using the object‐processing paradigm to investigate this possibility (Ishikawa et al. 2019). Results revealed superior encoding of a gaze‐cued object in 10‐ to 12‐months‐olds in an “initiating joint attention” condition, which simulated infants' object choice. This tentatively supports the assumption that infants' initiation of joint attention to an object of their “own choice” enhances infants' object memory. However, since infants' attention was externally manipulated via the experimental design in this study, infants were lacking the opportunity to make a genuine free choice. Further research needs to address this limitation by studying the influence of infants' intrinsic object choice on their learning (see D. Schmidt 2024 for current investigation).

#### 2.7.2 Considering Infants' Emotion

Another perspective related to infants' active role in social interactions, is infants' *self‐experienced emotion*. Previous research has shown that increased arousal enhances gaze following (Ishikawa and Itakura 2019), state‐related physiological changes are associated with capacities fostering stimulus processing and recognition (e.g., sustained attention, Frick and Richards 2001), and infants' recognition performance is enhanced when their arousal state matches their arousal state during encoding (state‐dependent memory, Seehagen et al. 2021). Findings regarding the effect of infants' emotional valence on learning are inconsistent. In a study by Nachman, Stern, and Best (1986), 7‐month‐olds expressing positive affect during familiarization with a puppet tended to look longer at this puppet in a subsequent paired‐preference test comparing the familiarized puppet with a novel one. When neutral affect was expressed during familiarization, however, infants showed a novelty preference. Conversely, a study by Rose, Futterweit, and Jankowski (1999) revealed a negative relation between positive affect and learning performance in 5‐ to 9‐month‐olds. More specific evidence from infants' learning in *social interaction* contexts suggest that infants react with positive emotions (expressed via smiling) when they successfully initiated a joint attention moment with others (Venezia et al. 2004; Venezia Parlade et al. 2009). Since infants' initiation of joint attention may foster their object encoding (Ishikawa et al. 2019), it is possible that infants' positive affect mediated their encoding success (Nachman, Stern, and Best 1986). How *negative* affect influences infants' information processing remains unclear. Future studies need to systematically capture variation in infants' emotional states during processing and recognition and relate it to their performance in the recognition test, for example, by using facial electromyography or pupillometry (Kaiser et al. 2017; Michel et al. 2021; D. Schmidt 2024).

#### 2.7.3 Capturing Human Social Learning Comprehensively

A growing body of literature, initially driven by cross‐cultural researchers, emphasizes the importance of adopting more inclusive perspectives on social learning to account for the diversity in the ways in which infants interact with and learn from others. While traditional studies on social learning have primarily focused on interaction settings characterized by high levels of infant‐directed communication and eye contact, as predominant in the Global North (Keller 2022), a more comprehensive understanding of social learning requires considering a wider range of learning settings. To fully uncover the mechanisms through which early social learning occurs, researchers have, for example, highlighted the relevance of studying observational learning (Shneidman and Woodward 2016), physical modalities of interpersonal connectedness (e.g., Barnett et al. 2022; Botero 2016) or group settings beyond the dyad (e.g., Keller, Decker, and Döge 2019; Moreland 2010).

A study by Thiele et al. (2021) demonstrates the potential of the object‐processing paradigm for expanding our understanding of infants' social learning beyond the traditional focus. Identical to interaction situations when directly addressed by an adult, 9‐month‐old German infants showed enhanced object encoding when merely observing joint attention interactions between third parties (see also Thiele et al. 2023). The authors concluded that, even in a context like urban Germany, where pedagogical forms of social learning are typically prevalent, infants show foundational abilities for observational learning during the first year of life. Given the strength of the paradigm to meticulously examine the influence of fine‐grained factors on infants' object memory when applied in carefully controlled designs, it offers a promising tool for exploring further understudied learning settings. Moreover, the simplicity and adaptability of the paradigm make it suitable for doing so in various testing environments, including remote field settings. This provides methodological opportunities for studying the ontogeny of human social learning more comprehensively, including children growing up in communities that remain underrepresented in psychological research (Nielsen et al. 2017).

## 3 Critical Evaluation

The object‐processing paradigm has been proven to be a valuable method for studying infants' object memory in social contexts. However, despite its contributions and potential, it should not be overlooked that there are several open questions and limitations of the paradigm warranting future research. In addition to the constraints outlined previously, one crucial avenue for future research concerns the paradigm's reliance on the novelty preference assumption. This assumption, which underlies the interpretation of infants' memory responses, is closely tied to broader discussions regarding familiarity and novelty preferences in infancy research (e.g., Kosie et al. 2024). While a novelty preference in the object‐processing paradigm is interpreted as evidence of successful encoding, other outcomes, such as the absence of a preference, are less straightforward to interpret, especially when looking times serve as the dependent variable. For instance, the absence of a preference could indicate either a failure to perceive or process the object during encoding, a loss of information during retention, or a retrieval failure during the recognition test. Alternatively, it could reflect the successful encoding of both the target and the novel object. These possibilities represent conceptually fundamentally different outcomes that cannot be distinguished by the dependent measure of the paradigm, highlighting the challenges in interpreting the absence of a preference.

Surprisingly, a familiarity preference has not been reported in the context of any of the studies included in this review. Given the relationship between infants' information processing stage and their novelty and familiarity preferences, a familiarity preference could potentially indicate partial or incomplete encoding of the target object (Pascalis and de Haan 2003; Rose et al. 1982). Alternatively, as discussed in section “Methodological Variations and Considerations in the Object‐Processing Paradigm”, it might reflect intrinsic responses beyond mere memory, such as approach or avoidance tendencies (Becchio, Bertone, and Castiello 2008)—again, two conceptually distinct possibilities. The absence of a familiarity preference in previous studies may be attributed to the relatively narrow age ranges examined. Studies with children older than 12 months yield inconsistent results, questioning the paradigm's robustness for older age groups. Previous research has shown that infants' visual recognition memory becomes increasingly flexible in the second year compared to the first 2 years of life (Robinson and Pascalis 2004). Moreover, the shift from familiar to novel information is influenced by infants' processing speed, their age, and the timing and complexity of the task (Hunter and Ames 1988; see also Kosie et al. 2024). To effectively apply this paradigm beyond the first year of life, adjustments would be necessary, e.g., by using an individual‐controlled, data‐driven approach to determine the duration of the valid measurement phase during the recognition test (e.g., by investigating the time‐course of infants' novelty or familiarity preference), or by adjusting the timing of the encoding phase. Furthermore, given infant's limited and varying attention span, it must be generally considered that the number of test trials and, thus, the number of testing conditions per participant are limited in a within‐subjects design.

Another limitation of the paradigm lies in the depth of the learning response it measures. While it offers valuable insights into fundamental memory aspects of infant object learning, it is not designed to capture deeper, more meaningful learning outcomes. The very few existing evidence on the persistence of the effect suggests that it may be transient, not extending beyond the immediate experimental trial (Parise et al. 2007; Theuring, Gredebäck, and Hauf 2007). To investigate the sustainability of the memory traces built for a specific object, ERP responses toward the previously cued object compared to responses toward the previously not cued object would be more informative than looking times to both objects assessed in a paired‐preference setup. ERPs offer greater temporal resolution, capture relatively fast brain responses, and provide the possibility to differentiate distinct consecutive processes, such as enhanced attention (NC) or enhanced encoding effort (PSW). The previously found short‐term duration, however, does not necessarily indicate limitations in gaze as a learning facilitator but rather reflects the nature of the memory stage under investigation. Focusing on the initial stages of the visual recognition memory, that is, building an unconsolidated memory trace, the paradigm does not measure the long‐term storage of memory traces requiring post‐encoding processes of stabilization and integration (Bauer et al. 2011). While this limitation restricts drawing conclusions about social learning outcomes, it is a key strength when applied to preverbal infants who have not yet developed targeted motor skills. By examining fundamental memory processes in infancy, before the development of complex cognitive and motor abilities, researchers can gain insights into the foundational building blocks of learning. These early processes may underlie more complex forms of object‐related knowledge and learning, such as the acquisition of culturally relevant information like functions, labels, or ritualistic actions. Studies with older children have shown that eye contact, within joint attention interactions, influences complex learning outcomes, including the learning of object‐related words, actions, and tool‐use functions (Hirotani et al. 2009; Matheson, Moore, and Akhtar 2013; Sage and Baldwin 2011). This suggests that the infants' responses in the object processing paradigm may represent precursors to these later abilities or reflect shared underlying mechanisms.

While the paradigm enables the investigation of this crucial early step in visual object processing, the studies included in this review offer a relatively limited understanding of the *kinds* of visual object features being processed. This is not necessarily due to limitations of the paradigm, but rather due to previous object choices often just described as “toys” with little explicit descriptions of their visual properties or criteria used for matching target and novel object (see columns “kinds of objects”, “general criteria for object matching” and “criteria for object pairs” in the OSF table). Future research could systematically investigate the encoding of specific object features by carefully selecting and manipulating object pairs based on their visual characteristics (e.g., showing an object of the same or different/novel semantic category, Needham, Dueker, and Lockhead 2005). Within such an approach, pupillometry could be a promising measure of infants' mental effort during the recognition phase (Marshall 2002). For example, object pairs perceived as more similar might result in greater pupil dilation than dissimilar pairs. Studies using a version of the violation‐of‐expectation paradigm have been shown to be useful in studying infants' encoding of specific object features in social situations involving gaze cues. These studies demonstrated the importance of specific object features (identity‐relevant object features over transient location), especially in the context of early cultural learning (Okumura, Kobayashi, and Itakura 2016; Yoon, Johnson, and Csibra 2008). However, the replication crisis and inconsistent findings in detailed result patterns (Silverstein et al. 2019; see also discussion in Thiele et al. 2023) highlight the need for further investigation, for example, exploring pupil dilation as an alternative measure of expectancy violation (Margoni, Surian, and Baillargeon 2023). Employing the here reviewed object‐processing paradigm to explore these empirical questions could contribute to ongoing discussions and a more comprehensive understanding of infant visual perception.

Importantly, this review is limited by its focus on published studies. Due to the publication bias for positive test results in psychological research more generally (Kühberger, Fritz, and Scherndl 2014), this leads to a biased perspective making it difficult to assess the robustness of methodological details and identify conditions under which the paradigm may not be effective. To address this problem, it requires incentivizing and facilitating the publication of null results, embracing open‐science practices, and supporting large‐scale replication efforts as has been successfully implemented in other domains such as the habituation paradigm (Kucharský et al. 2024), online eye‐tracking paradigms (Zaadnoordijk et al. 2021), or preference‐based paradigms more broadly (Kosie et al. 2024, ManyBabies). This would enable an even more comprehensive understanding of the strengths and limitations of the object‐processing paradigm, and of the details to consider when applying it.

## 4 Conclusions

The past two decades of research applying the object‐processing paradigm have provided us with substantial empirical evidence regarding infants' social learning. The paradigm has proven to be a powerful tool for disentangling the influence of fine‐grained factors on infants' object memory. However, to fully exploit its potential, future research must delve deeper into the intricacies of infant memory processes and establish rigorous methodological standards. This will be essential for optimizing the paradigm's application—for advancing our understanding of infant social learning and beyond.

## Author Contributions

**Christine Michel:** conceptualization, data curation, formal analysis, investigation, project administration, writing–original draft, writing–review and editing. **Maleen Thiele:** conceptualization, data curation, formal analysis, investigation, project administration, writing–original draft, writing–review and editing.

## Conflicts of Interest

The authors declare no conflicts of interest.

## Supporting information

Supporting Information S1

## Data Availability

The data summarizing the studies reviewed in this article are openly available on the Open Science Framework (OSF). Link to OSF project repository: https://osf.io/5p4w8/. Direct link to OSF table: https://osf.io/ysrje (please download.xlsx file for correct formatting).
